# Influencing factors on attendance in cervical cancer screening among women with diabetes in Hungary: a cross-sectional study using European Health Interview Surveys 2009-2019

**DOI:** 10.3389/fonc.2025.1501654

**Published:** 2025-04-29

**Authors:** Eszter Faludi Vargáné, Amr Sayed Ghanem, Chau Minh Nguyen, Jenifer Pataki, Gergő József Szőllősi, Attila Csaba Nagy

**Affiliations:** ^1^ Department of Integrative Health Sciences, Faculty of Health Sciences, University of Debrecen, Debrecen, Hungary; ^2^ Department of Health Informatics, Faculty of Health Sciences, University of Debrecen, Debrecen, Hungary; ^3^ Coordination Center for Research in Social Sciences, Faculty of Economics and Business, University of Debrecen, Debrecen, Hungary; ^4^ Gottsegen György National Cardiovascular Center, Budapest, Hungary

**Keywords:** cervical cancer, cervical cancer screening, attendance, diabetes mellitus, European Health Interview Survey

## Abstract

**Introduction:**

With this study, we examined the participation in cervical cancer screening among women with diabetes and the influencing factors of attendance.

**Methods:**

Data from the European Health Interview Surveys in Hungary (2009, 2014, 2019) were analyzed with multivariate and multiple logistic regressions.

**Results:**

A higher level of education (OR=2.56, 95% CI: 1.03-6.33 in the case of secondary level in 2014; and OR=3.09, 95% CI: 1.17-8.13 in the case of tertiary level in 2019, OR= 2.24, 95% CI: 1.12-4.46 in the case of tertiary level in the pooled data), a perceived good economic situation (OR=2.31, 95% CI: 1.30-4.09 in the pooled data), participation in breast cancer screening (OR= 5.41, 95% CI: 3.49-8.38 in the pooled data), and social support (OR= 2.04 95% CI: 1.03-4.03 in 2019) have a positive effect on participation in screening. Taking prescription drugs (OR= 0.31 95% CI: 0.12-0.83, in the pooled data), lower economic status (OR=0.25 95% CI:0.07-0.88, in 2009) and worse perceived health (OR= 0.20, 95% CI: 0.06-0.64 in 2014) can be considered factors with a negative effect.

**Conclusion:**

This study identified groups with low participation rates and made it clear that those groups with unfavorable health factors (bad financial status, bad perceived health, taking prescription drugs) participate the least in screening.

## 1 Introduction

### 1.1 The connection between diabetes mellitus and cervical cancer

Cervical cancer is one of the leading causes of cancer death among women (1). According to the GLOBOCAN database, in 2022, there were an estimated 660–000 cervical cancer new cases and 350–000 deaths globally (2). Despite the fact that this is a well-curable tumor, the cervical cancer’s mortality was on the sixth position in 2020 in the world (3). The risk factors of the cervical cancer are mostly generally known, they include HPV infection, smoking, sexual behavior and oral contraceptive use (4, 5). However, it is a less well-known fact that obesity and diabetes also increase the risk of cervical cancer’s development (6). According to a study, diabetes increases the likelihood of developing cervical cancer by twofold (7). As diabetes mellitus is a common metabolic disease, a lot of women has a higher risk according to cervical cancer. According to the World Health Organization’s (WHO) 2024 report, the number of people living with diabetes rose from 200 million in 1990 to 830 million in 2022 (8). The gender distribution is equal, and the incidence peaks at around 55 years of age (9). Studies proved, that there is a causal association between genetic predisposition to type 2 diabetes and many cancers, like cervical cancer (10, 11). Possible associations between the two conditions include various metabolic abnormalities such as obesity, hyperglycemia, hyperinsulinemia stimulation of the IGF-1 (insulin growth factor-1) axis, and increased cytokine production (12). Other studies have described that diabetes is associated with a higher incidence of other diseases of the cervix (for example, vaginitis, cervicitis, HPV infection, and malignant tumors). Certain genes showed a positive correlation with both conditions (diabetes mellitus and cervical pathologies), such as COLL11A2P1 (beta 0.06), INS-IGF2, and TTC 723 (13). In addition to the fact that diabetes poses a risk for some gynecological malignancies, cervical cancer’s prognosis is worse by patients with diabetes (14). Obese women with cervical cancer, especially in postmenopausal period, had a significantly higher mortality related to non-obese women (15), but obesity might increase the incidence of cervical cancer among premenopausal women too (16).

### 1.2 Cervical cancer screening in Hungary

In Hungary, on the basis of Decree 51/1997 (XII.18) NM, cervical cancer screening has been carried out in an organized form - every 3 years - since 2003 for women aged 25–65 who are eligible according to the National Health Insurance Fund Management Register. Women between the ages of 25 and 65 who have not undergone cervical cancer screening publicly funded by the National Health Insurance Fund within three years from the scheduled date of the screening will receive an invitation by mail to a screening, which they can attend at the gynecology clinic or at the nurse’s office (17).

### 1.3 Cervical cancer screening among women with diabetes

In overweight or obese women, the risk of the disease is also increased by the fact that adequate sample collecting equipment is not always available, so they have a higher chance of underdiagnosis during screening (18). For this reason, it would be much more important for women with diabetes to attend screenings in order to prevent these diseases and improve the survival rate. Especially considering the fact that this is the only cancer that could be completely prevented by vaccination, as well as treatment after early diagnosis (19). However, some studies showed, that if a patient has diabetes it was associated with significantly lower likelihood to participate in cervical cancer screening, relative to not having diabetes (20–23). In addition to being a risk factor for cervical cancer, a study’s measurements showed that survival rates were worse for women with both diabetes and cervical cancer compared to those with cervical cancer without diabetes (24).

Our aim was to identify the most vulnerable groups in Hungary among women with diabetes, who are least likely to undergo cervical cancer screening, in order to explore the target groups of health promotion programs.

## 2 Methods

The study is based on data from the European Health Interview Survey (EHIS) of 2009, 2014 and 2019, the basis of which was developed by Eurostat in compliance with a legal obligation. The EHIS uses a standardized questionnaire that contains the following main groups of questions: health status, use of the health care system, drug use, reasons for refusing health care, opinion about the health care system, use of preventive care, general well-being, factors affecting health, care, demographic data. The questionnaire also contains separate groups of questions for children. Its main purpose is to collect data for European health indicators (25, 26). The EHIS provides age data in three categories: 15-34, 35-64, and 65+, making it impossible to define a precise eligibility cutoff (e.g., 25–65 years) in alignment with national screening guidelines. Given this limitation, we used a binary classification: below 65 years and 65+ years, ensuring that the analysis captures the vast majority of screening-eligible women (i.e., those below 65) while distinguishing those who may have aged out of routine screening recommendations. Although some younger women (15-24) were included in the <65 category, they represent a minority, and their inclusion does not substantially affect overall trends, as participation in screening is expected to be low in this subgroup. This categorization balances data constraints with epidemiological relevance, allowing for meaningful comparisons while adhering to the best available classification method given the dataset structure.

In Hungary, the respondents were selected based on two-stage stratified sampling according to county and settlement size, the sampling method was developed by Hungarian Central Statistical Office (27). The total sample included 16,480 persons aged 15 and over living in private households, of which 8,910 were women, of whom 700 were women with diabetes. The outcome variable of the study was participation in cervical cancer screening among women with diabetes. In the questionnaire “When was the last time you had a gynecological cervical cancer screening?” question related to this: the five answer options were divided into two categories: the category of having participated in screening for less than 2 years and the category of having participated in screening for more than 2 years. Data on diabetes were obtained from answers to the question on diabetes, in the chronic diseases block.

As influencing factors age (≥65, <65) marital status (single/in a relationship), level of education (primary/secondary/tertiary), type of occupation (employed/unemployed), area of residence (urban/rural), regions (Central Hungary/Southern Great Plain/Southern Transdanubia/Central Transdanubia/Western Transdanubia/Northern Great Plain/Northern Hungary) financial situation (average/good/bad), income quintiles (first/second/third/fourth/fifth), BMI overweight + obese/normal), smoking status (smoker/non-smoker), alcohol consumption (drinker/non-drinker), self-perceived health (average/good/bad), hypertension (yes/no), hypercholesterolemia (yes/no), cardiovascular disease (yes/no), depression (yes/no), mental illnesses (yes/no), last visit to the dental office (more than a year ago/less than a year ago), taking prescription drugs (yes/no), taking supplements (yes/no) and breast cancer screening attendance (more than 2 years/within the past 2 years) were included. Variables with excessive missing values were excluded from the analysis, and missing data in retained variables were handled using listwise deletion.

### 2.1 Statistical methods

The statistical analysis applied weighted methods throughout to ensure representativeness and accuracy given the survey design. Weighted proportions were calculated to describe the distribution of screening participation and predictor variables across the survey years, and weighted chi-square tests were conducted to assess associations between categorical predictors and cervical cancer screening participation while adjusting for sampling weights. For inferential analysis, weighted multiple logistic regression models were developed to estimate adjusted odds ratios (ORs) with 95% confidence intervals (CIs), controlling for potential confounders while accounting for survey weights. Bootstrapping with 1,000 iterations was applied to estimate robust confidence intervals for screening participation rates. Sensitivity analysis was conducted, and the model with the lowest Akaike Information Criterion and Bayesian Information Criterion was chosen as the final model. To ensure validity, the Hosmer-Lemeshow goodness-of-fit test was performed to assess model calibration, and multicollinearity was tested using the Variance Inflation Factor (VIF), confirming no collinearity concerns. Model discrimination was evaluated using the area under the receiver operating characteristic curve (AUC-ROC), and a confusion matrix was generated to assess classification accuracy, including sensitivity, specificity, positive predictive value, and negative predictive value. The result was considered significant if the p value was below 0.05. STATA IC Version 17.0 software was used for statistical analysis. In addition to these, we also compared the results of each year.

## 3 Results

The sample contains 700 women with diabetes, in the annual distribution below: 164 in 2009, 250 in 2014, and 285 in 2019. The age distribution of women with diabetes was as follows: 4.57% of the respondents belonged to the age group of 15-34, 40.57% to the age group of 35–64 and 54.86% to the age group of 65+.

### 3.1 Characteristics of participants in 2009, 2014 and 2019 and pooled data

Regarding the age group categories, there was a significant difference in screening participation in all three years, with the oldest age group’s attendance rate was the lowest. The participation rates in cervical cancer screening for individuals under 65 years of age were as follows in 2009, 2014, and 2019, as well as the overall data: 68.94%, 68.73%, 64.74%, and 67.18%. The level of education also had a significant influencing effect of the attendance: in 2014, in 2019 and, in the pooled data the lowest education level group showed the lowest rate, in the case of family status, the same result was obtained for single people. Occupation has significant influence on attendance rate in all three years, employed respondents attended screening in a significantly higher proportion in each examined year, as well as a better financial situation also increases the participation rate.

Examining the lifestyle factors, it can be concluded that a significantly lower proportion of alcohol drinkers took part in screening in 2009 and 2019, while in the case of smokers, a significant difference in screening can only be observed in 2019. The group who considered their health to be good used the screening test more often, and in 2014 and in 2019 the same could be said for those living without hypertension and CVD. In the case of using other health services - such as a dental examination, participating in a breast screening - the respondents also took part in cervical cancer screening significantly more often in each examined year. Regarding the use of medication, it can be said that the participation rate was lower in the case of those taking prescription medication, while it was higher in the case of OTC medication use (**Table 1**).

**Table 1:** Characteristics of participants in 2009, 2014, 2019 and pooled data.

Characteristics		2009			2014			2019			Pooled		
		Did not attend n (%)	Attended n (%)	P-value	Did not attend n (%)	Attended n (%)	P-value	Did not attend n (%)	Attended n (%)	P-value	Did not attend n (%)	Attended n (%)	P-value*
Age categories	≥65	50 (67.81%)	28 (31.06%)	**0.001**	105 (71.98%)	30 (31.27%)	**0.001**	119 (77.89%)	52 (35.26%)	**<0.001**	274 (73.05%)	110 (32.82%)	**<0.001**
	<65	25 (32.19%)	61 (68.94%)		42 (28.02%)	73 (68.73%)		34 (22.11%)	80 (64.74%)		101 (26.95%)	214 (67.18%)	
Relationship status	Single	45 (60.53%)	44 (44,80%)	0.056	92 (62.90%)	44 (46.10%)	**0.012**	97 (64.06%)	55 (40.04%)	**0.001**	234 (62.74%)	143 (43.30%)	**<0.001**
	In a relationship	29 (39.47%)	46 (55.20%)		55 (37.10%)	57 (53.90%)		53 (35.94%)	74 (59.96%)		137 (37.26%)	177 (56.70%)	
Educational status	Primary	57 (74.24%)	62 (67.93%)	0.691	109 (73.78%)	51 (46.85%)	**0.001**	94 (64.55%)	60 (41.48%)	**0.001**	260 (70.66%)	173 (50.65%)	**<0.001**
	Secondary	12 (18.18%)	21 (23.65%)		29 (20.59%)	35 (34.53%)		42 (25.93%)	46 (36.02%)		83 (21.88%)	102 (32.05%)	
	Tertiary	6 (7.58%)	7 (8.42%)		9 (5.63%)	17 (18.62%)		17 (9.52%)	26 (22.50%)		32 (7.46%)	50 (17.30%)	
Employment status	Unemployed	71 (94.27%)	71 (77.92%)	**0.007**	130 (89.54%)	72 (72.69%)	**0.001**	136 (88.88%)	82 (59.36%)	**0.001**	337 (90.44%)	225 (68.78%)	**<0.001**
	Employed	4 (5.73%)	19 (22.08%)		17 (10.46%)	31 (27.31%)		17 (11.12%)	50 (40.64%)		38 (9.56%)	100 (31.22%)	
Area of residence	Rural	17 (20.15%)	26 (27.29%)	0.289	55 (35.02%)	30 (24.54%)	0.078	47 (30.65%)	41 (28.23%)	0.664	119 (29.93%)	97 (26.81%)	0.37
	Urban	58 (79.85%)	64 (72.71%)		92 (64.98%)	73 (75.46%)		106 (69.35%)	91 (71.77%)		256 (70.07%)	228 (73.19%)	
Regions	Central Hungary	15 (25.20%)	23 (30.72%)	0.113	41 (30.63%)	27 (31.71%)	**0.013**	55 (35.10%)	40 (30.48%)	0.547	111 (30.90%)	90 (30.93%)	0.264
	Southern Great Plain	11 (13.47%)	12 (12.75%)		13 (8.34%)	17 (15.93%)		16 (11.74%)	16 (10.74%)		40 (10.76%)	45 (12.93%)	
	Southern Transdanubia	7 (8.76%)	9 (8.93%)		12 (7.72%)	19 (15.26%)		11 (7.59%)	14 (10.65%)		30 (7.92%)	42 (11.60%)	
	Northern Great Plain	8 (9.55%)	14 (14.27%)		17 (9.73%)	16 (13.83%)		27 (16.42%)	16 (12.20%)		52 (12.03%)	46 (13.30%)	
	Central Transdanubia	10 (11.48%)	6 (6.46%)		18 (12.05%)	10 (9.57%)		19 (13.73%)	16 (12.09%)		47 (12.50%)	32 (9.71%)	
	Northern Hungary	21 (26.86%)	13 (12.32%)		27 (18.22%)	5 (5.14%)		13 (8.77%)	22 (16.21%)		61 (16.98%)	40 (11.65%)	
	Western Transdanubia	3 (4.68%)	13 (14.55%)		19 (13.31%)	9 (8.55%)		12 (6.65%)	8 (7.64%)		34 (8.91%)	30 (9.88%)	
Financial status	Average	40 (50.22%)	41 (45.89%)	0.316	87 (59.20%)	58 (54.50%)	0.095	99 (65.71%)	72 (56.70%)	0.212	226 (59.28%)	171 (52.92%)	**0.011**
	Good	2 (2.47%)	5 (7.98%)		13 (8.87%)	15 (18.58%)		26 (17.67%)	35 (26.76%)		41 (10.37%)	55 (18.81%)	
	Bad	33 (47.31%)	42 (46.13%)		47 (31.94%)	29 (26.93%)		25 (16.62%)	20 (16.54%)		105 (30.35%)	91 (28.27%)	
Income quintiles	First	12 (17.69%)	16 (19.34%)	0.347	66 (45.35%)	33 (30.74%)	0.174	30 (20.54%)	29 (23.19%)	0.179	108 (30.04%)	78 (24.46%)	0.231
	Second	11 (15.40%)	26 (27.99%)		30 (19.62%)	24 (22.43%)		50 (33.74%)	34 (23.69%)		91 (23.55%)	84 (24.51%)	
	Third	16 (24.25%)	15 (17.29%)		25 (15.47%)	17 (15.49%)		35 (22.48%)	24 (18.29%)		76 (20.02%)	56 (17.13%)	
	Fourth	23 (26.06%)	18 (18.93%)		15 (11.71%)	17 (19.14%)		26 (15.94%)	32 (25.37%)		64 (16.63%)	67 (21.60%)	
	Fifth	13 (16.60%)	15 (16.44%)		11 (7.86%)	12 (12.19%)		12 (7.30%)	13 (9.46%)		36 (9.76%)	40 (12.29%)	
BMI	Overweight/obese	62 (81.82%)	74 (80.21%)	0.811	112 (76.02%)	80 (76.33%)	0.957	121 (79.79%)	105 (77.62%)	0.683	295 (78.75%)	259 (77.95%)	0.815
	Normal	13 (18.18%)	16 (19.79%)		31 (23.98%)	23 (23.67%)		31 (20.21%)	24 (22.38%)		75 (21.25%)	63 (22.05%)	
Smoking status	Smoker	13 (18.76%)	20 (26.34%)	0.289	17 (10.58%)	16 (14.87%)	0.315	20 (12.31%)	29 (24.34%)	**0.012**	50 (13.13%)	65 (21.90%)	**0.004**
	Non-smoker	61 (81.24%)	67 (73.66%)		130 (89.42%)	87 (85.13%)		133 (87.69%)	103 (75.66%)		324 (86.87%)	257 (78.10%)	
Alcohol consumption	Drinker	20 (25.50%)	41 (46.38%)	**0.009**	64 (44.94%)	49 (49.99%)	0.449	58 (37.74%)	68 (51.13%)	**0.031**	142 (37.77%)	158 (49.45%)	**0.003**
	Non-drinker	55 (74.50%)	46 (53.62%)		83 (55.06%)	54 (50.01%)		95 (62.26%)	64 (48.87%)		233 (62.23%)	164 (50.55%)	
Self-perceived health	Average	29 (37.46%)	37 (38.67%)	0.483	59 (39.26%)	51 (48.31%)	**0.002**	84 (54.60%)	58 (43.42%)	**0.007**	172 (44.20%)	146 (43.60%)	**<0.001**
	Good	4 (5.25%)	9 (10.16%)		22 (14.98%)	27 (27.39%)		24 (15.19%)	39 (31.74%)		50 (12.72%)	75 (24.23%)	
	Bad	42 (57.29%)	44 (51.18%)		66 (45.76%)	25 (24.29%)		45 (30.20%)	33 (24.83%)		153 (43.08%)	102 (32.17%)	
Hypertension	No	11 (14.11%)	16 (19.77%)	0.365	27 (18.67%)	24 (25.52%)	0.221	34 (21.98%)	38 (33.04%)	**0.05**	72 (18.73%)	78 (26.93%)	**0.015**
	Yes	64 (85.89%)	74 (80.23%)		120 (81.33%)	79 (74.48%)		119 (78.02%)	94 (66.96%)		303 (81.27%)	247 (73.07%)	
Hypercholesterolemia	No	43 (58.80%)	56 (63.20%)	0.582	91 (61.97%)	68 (67.00%)	0.431	83 (56.27%)	80 (62.76%)	0.295	217 (59.24%)	204 (64.23%)	0.197
	Yes	31 (41.20%)	34 (36.80%)		56 (38.03%)	35 (33.00%)		67 (43.73%)	48 (37.24%)		154 (40.76%)	117 (35.77%)	
Cardiovascular disease	No	29 (39.39%)	45 (49.90%)	0.198	89 (61.09%)	65 (64.76%)	0.566	86 (55.95%)	90 (68.83%)	**0.032**	204 (54.09%)	200 (62.20%)	**0.038**
	Yes	46 (60.61%)	45 (50.10%)		58 (38.91%)	38 (35.24%)		67 (44.05%)	42 (31.17%)		171 (45.91%)	125 (37.80%)	
Depression	No	61 (80.09%)	67 (74.47%)	0.416	129 (87.42%)	87 (84.55%)	0.534	139 (91.48%)	123 (93.60%)	0.503	329 (87.07%)	277 (85.36%)	0.531
	Yes	14 (19.91%)	23 (25.53%)		18 (12.58%)	16 (15.45%)		13 (8.52%)	9 (6.40%)		45 (12.93%)	48 (14.64%)	
Mental illness	No	71 (94.00%)	82 (91.25%)	0.526	133 (91.25%)	97 (93.80%)	0.48	131 (86.57%)	113 (87.77%)	0.772	335 (90.28%)	292 (90.67%)	0.866
	Yes	4 (6.00%)	8 (8.75%)		14 (8.75%)	6 (6.20%)		21 (13.43%)	16 (12.23%)		39 (9.72%)	30 (9.33%)	
Last visit to the dental office	More than a year ago	58 (76.26%)	62 (66.42%)	0.2	113 (77.15%)	60 (59.83%)	**0.005**	106 (69.90%)	77 (56.56%)	**0.028**	277 (74.41%)	199 (60.38%)	**<0.001**
	Less than a year ago	16 (23.74%)	28 (33.58%)		34 (22.85%)	43 (40.17%)		46 (30.10%)	54 (43.44%)		96 (25.59%)	125 (39.62%)	
Taking prescription drugs	No	4 (4.60%)	5 (6.79%)	0.561	1 (0.55%)	7 (7.59%)	**0.001**	7 (4.80%)	6 (6.60%)	0.568	12 (3.01%)	18 (6.96%)	**0.022**
	Yes	71 (95.40%)	85 (93.21%)		146 (99.45%)	96 (92.41%)		146 (95.20%)	126 (93.40%)		363 (96.99%)	307 (93.04%)	
Taking supplements	No	43 (56.47%)	56 (63.58%)	0.372	73 (50.08%)	44 (40.22%)	0.137	56 (37.34%)	43 (33.24%)	0.495	172 (47.15%)	143 (44.01%)	0.428
	Yes	32 (43.53%)	34 (36.42%)		74 (49.92%)	59 (59.78%)		97 (62.66%)	89 (66.76%)		203 (52.85%)	182 (55.99%)	
Breast cancer screening attendance	More than 2 years	39 (68.25%)	18 (23.79%)	**<0.001**	113 (77.17%)	22 (22.19%)	**0.001**	111 (73.28%)	51 (41.68%)	**<0.001**	263 (73.99%)	91 (30.90%)	**<0.001**
	Within the past 2 years	20 (31.75%)	56 (76.21%)		34 (22.83%)	81 (77.81%)		41 (26.72%)	81 (58.32%)		95 (26.01%)	218 (69.10%)	

*Pearson’s chi-squared test. Statistically significant values are shown in bold.

### 3.2 Multiple logistic regression models

In multiple regression analysis, adjusting for age, we found a significant difference in the attendance rates of cervical cancer screening rates in the year 2014, according to the reference year 2009, a decreasing trend can be observed. A higher level of education (OR=2.56, 95% CI: 1.03-6.33 in the case of secondary level in 2014; and OR=3.09, 95% CI: 1.17-8.13 in the case of tertiary level in 2019, OR= 2.24, 95% CI: 1.12-4.46 in the case of tertiary level in the pooled data), a perceived good economic situation (OR=2.31, 95% CI: 1.30-4.09 in the pooled data), participation in breast cancer screening (OR= 5.41, 95% CI: 3.49-8.38 in the pooled data), and social support (OR= 2.04 95% CI: 1.03-4.03 in 2019) have a positive effect on participation in screening. Taking prescription drugs (OR= 0.31 95% CI: 0.12-0.83, in the pooled data), lower economic status (OR=0.25 95% CI:0.07-0.88, in 2009) and worse perceived health (OR= 0.20, 95% CI: 0.06-0.64 in 2014) can be considered factors with a negative effect. Women under age 65 years old have significantly higher odds to attend the cervical cancer screening according to the pooled data (p<0.001). (**Table 2**).

**Table 2:** Possible influencing factors of attendances on cervical screening programs.

Characteristics		2009		2014		2019		Pooled	
		OR [95% CI]	P value	OR [95% CI]	P value	OR [95% CI]	P value	OR [95% CI]	P value
Year of survey	2009 (ref)								
	2014							**0.45 [0.24-0.82]**	**0.01**
	2019							0.69 [0.37-1.30]	0.252
Age categories	≥65 (ref)								
	<65	1.83 [0.92-3.62]	0.085	1.66 [0.92-3.00]	0.093	**2.26 [1.37-3.71]**	**0.001**	**3.24 [1.94-5.39]**	**<0.001**
Relationship status	Single (ref)								
	In a relationship	0.67 [0.24-1.86]	0.441	1.05 [0.39-2.85]	0.925	**2.04 [1.03-4.03]**	**0.04**	1.39 [0.93-2.09]	0.111
Educational status	Primary (ref)								
	Secondary	0.26 [0.06-1.08]	0.064	**2.56 [1.03-6.33]**	**0.043**	1.68 [0.72-3.92]	0.231	1.29 [0.78-2.13]	0.318
	Tertiary	0.85 [0.13-5.69]	0.867	2.59 [0.68-9.86]	0.162	**3.09 [1.17-8.13]**	**0.022**	**2.24 [1.12-4.46]**	**0.022**
Employment status	Unemployed (ref)								
	Employed	4.18 [0.71-24.65]	0.114	0.44 [0.13-1.50]	0.191	1.07 [0.37-3.15]	0.896	1.44 [0.79-2.60]	0.233
Area of residence	Rural (ref)								
	Urban	0.71 [0.13-3.71]	0.68	1.11 [0.44-2.77]	0.827	1.84 [0.83-4.06]	0.131	1.25 [0.79-1.98]	0.333
Regions	Central Hungary (ref)								
	Southern Great Plain	0.43 [0.07-2.79]	0.374	1.40 [0.36-5.42]	0.627	1.51 [0.34-6.66]	0.585	1.00 [0.48-2.09]	0.996
	Southern Transdanubia	0.24 [0.02-2.37]	0.217	1.60 [0.35-7.23]	0.54	1.70 [0.52-5.62]	0.382	1.31 [0.62-2.75]	0.483
	Northern Great Plain	0.63 [0.06-7.14]	0.708	1.32 [0.35-4.98]	0.679	0.77 [0.31-1.89]	0.561	1.06 [0.57-1.96]	0.864
	Central Transdanubia	0.29 [0.06-1.36]	0.115	1.89 [0.48-7.45]	0.364	1.18 [0.40-3.47]	0.764	1.07 [0.52-2.18]	0.857
	Northern Hungary	1.35 [0.11-16.39]	0.815	**0.07 [0.02-0.28]**	**<0.001**	3.22 [0.90-11.48]	0.071	0.64 [0.33-1.25]	0.188
	Western Transdanubia	1.35 [0.11-16.39]	0.815	1.45 [0.23-8.98]	0.691	**3.85 [1.07-13.90]**	**0.039**	1.59 [0.69-3.65]	0.275
Financial status	Average (ref)								
	Good	2.27 [0.25-20.74]	0.465	**4.50 [1.35-14.99]**	**0.015**	1.75 [0.73-4.20]	0.212	**2.31 [1.30-4.09]**	**0.004**
	**Bad**	**0.25 [0.07-0.88]**	**0.032**	1.37 [0.53-3.54]	0.508	0.93 [0.38-2.32]	0.881	0.77 [0.46-1.29]	0.316
Income quintiles	First (ref)								
	Second	3.67 [0.51-26.33]	0.194	1.33 [0.38-4.70]	0.653	0.81 [0.29-2.27]	0.681	0.92 [0.50-1.69]	0.786
	Third	0.81 [0.12-5.70]	0.832	0.86 [0.25-2.99]	0.818	1.08 [0.39-3.01]	0.879	0.73 [0.40-1.33]	0.301
	Fourth	0.29 [0.05-1.73]	0.175	1.06 [0.25-4.38]	0.94	1.07 [0.35-3.31]	0.905	0.57 [0.29-1.12]	0.103
	Fifth	0.59 [0.07-4.76]	0.615	0.29 [0.07-1.17]	0.081	0.43 [0.09-2.04]	0.287	0.44 [0.20-1.01]	0.053
BMI	Overweigh/obese (ref)								
	Normal	1.98 [0.36-10.77]	0.428	1.61 [0.63-4.12]	0.323	1.01 [0.50-2.04]	0.979	1.16 [0.69-1.94]	0.571
Smoking	Smoker (ref)								
	Non-smoker	1.25 [0.24-6.52]	0.791	0.90 [0.24-3.35]	0.88	0.87 [0.34-2.23]	0.774	1.13 [0.63-2.05]	0.682
Alcohol consumption	Drinker (ref)								
	Non-drinker	0.84 [0.29-2.43]	0.75	1.28 [0.54-3.06]	0.574	0.84 [0.41-1.73]	0.634	1.03 [0.68-1.57]	0.875
Self-perceived health	Average (ref)								
	Good	0.59 [0.06-6.13]	0.659	0.86 [0.32-2.29]	0.76	1.57 [0.69-3.58]	0.285	1.31 [0.72-2.38]	0.378
	Bad	0.78 [0.23-2.70]	0.698	**0.20 [0.06-0.64]**	**0.007**	1.50 [0.66-3.43]	0.331	0.72 [0.43-1.21]	0.217
Hypertension	No (ref)								
	Yes	2.70 [0.49-14.69]	0.249	0.62 [0.21-1.80]	0.375	1.46 [0.62-3.44]	0.381	1.35 [0.79-2.32]	0.277
Hypercholesterolemia	No (ref)								
	Yes	0.60 [0.19-1.91]	0.38	0.72 [0.30-1.74]	0.47	0.74 [0.38-1.47]	0.394	0.78 [0.50-1.23]	0.287
Cardiovascular disease	No (ref)								
	Yes	1.25 [0.42-3.69]	0.689	2.02 [0.81-5.04]	0.13	1.51 [0.67-3.40]	0.32	1.40 [0.88-2.21]	0.151
Depression	No (ref)								
	Yes	0.64 [0.16-2.56]	0.522	1.77 [0.47-6.62]	0.394	0.37 [0.11-1.26]	0.112	0.94 [0.48-1.82]	0.844
Mental illness	No (ref)								
	Yes	3.86 [0.46-32.21]	0.21	0.98 [0.18-5.38]	0.982	1.08 [0.34-3.42]	0.894	1.42 [0.67-3.04]	0.362
Last visit to the dental office	More than a year ago (ref)								
	Less than a year ago	0.52 [0.16-1.70]	0.278	1.87 [0.83-4.20]	0.131	0.99 [0.50-1.94]	0.976	1.04 [0.66-1.62]	0.878
Taking prescription drugs	No (ref)								
	Yes	0.21 [0.02-2.18]	0.187	**0.03 [0.00-0.35]**	**0.006**	0.63 [0.16-2.50]	0.508	**0.31 [0.12-0.83]**	**0.02**
Taking supplements	No (ref)								
	Yes	1.96 [0.62-6.25]	0.253	1.31 [0.57-3.03]	0.521	0.91 [0.45-1.84]	0.796	0.98 [0.63-1.51]	0.925
Breast cancer screening attendance	More than 2 years (ref)	0.06 [0.02-0.2]	**<0.001**	0.06 [0.02-0.14]					
	Within the past 2 years	**15.94 [4.96-51.21]**		**18.06 [6.98-46.70]**	**<0.001**	**3.30 [1.61-6.76]**	**0.001**	**5.41 [3.49-8.38]**	**<0.001**

Odds ratios and 95% confidence intervals of multiple logistic regression are reported. Statistically significant values are shown in bold.

## 4 Discussion

Our aim was to identify the influencing factors that are contribute to the attendance of cervical cancer screening among diabetic women.

The participation rate of women with diabetes is declining compared to the 2009 baseline.

Higher education, good economic situation, participation in breast cancer screening and social support have a positive effect on participation in screening. Taking prescription drugs, lower economic status, and poorer perceived health negatively affected participation in screening.

Compared to the reference year (2009), the attendance rate was lower in the other two examined years, the result was significant in 2014. Some studies also show this declining trend on attendance (28), others report increasing participation rates (29). The decline in participation rate can be explained by the introduction of HPV vaccination in Hungary, which happened in 2014.

Similar to other studies (30, 31), positive correlations were detected to screening attendance with the following factors: higher educational level, social support, better financial state. International recommendations no longer recommend cervical cancer screening for people older than 65, except in some special cases (32, 33). This can explain why the younger age group took part in the screening examination in greater proportion. Participation in breast cancer screening also shows a correlation with participation in cervical cancer screening in other countries (34).

Some factors decreased the odds of the attendance of cervical cancer screening, these were bad financial state and worse perceived health status and taking prescription drugs. These factors have also been identified as influencing factors in other studies (35, 36). The decreasing participation rate does not only affect women with diabetes, but is also characteristic of the entire female sample based on the data examined. According to another study analyzing participation data in Hungary, attendance in cervical cancer screening during the period 2008–2021 initially showed an increase, but from 2016 onwards a continuously decreasing trend can be observed (37). Based on OECD statistics, from 2011–2021 among the examined 31 countries, Hungary had the third lowest attendance rate in cervical cancer screening, only Poland and Costa Rica had lower numbers. The OECD average rate was 53%, in Hungary this number was only 26%, and the OECD found that the attendance rate decreased with 12% compared to the data from 2011 (38).

Reviewing Hungarian cervical cancer mortality data, HPV vaccination and cervical cancer treatment, there are still areas that must be improved in order to reach the 90-70–90 goal formulated by the World Health Organization, which includes the following:90% HPV vaccination coverage rate among girls by the age of 15; a 70% coverage of cervical cancer screening at ages 35 and 45 and treating 90% of women with precancer and managing 90% of women with invasive cancer. The WHO goal for the incidence of cervical cancer is lower than 4 new cases per 100–000 women, each year (39). In Hungary the HPV vaccination rate was 82% in 2021, the number of new cases was 24,7/100–000 women, and the 75% of 25-65-year-old women were screened in the last three years (40). The situation in Hungary is not favorable in terms of screening participation, the participation rate in cervical cancer screening tests was under 50% in 2006 and it has been decreasing over the years (in 2019 only 30.2% of 20–69 year olds appeared for screening) (41). According to the results of a meta-analysis, increasing participation in cervical cancer screening would be facilitated by the possibility of self-sampling and sending invitation letters and reminders to affected women (42).

Many factors have influence on attendance of screening programs. Socioeconomic status (SES) influences people’s health behavior, studies found that higher SES has positive effect on the participation of screening programs (43, 44). However, analyzing the individual components of socioeconomic status (educational level, marital status, financial status), the relationship is not so clear. There are components that clearly have a positive effect on screening participation, e.g. married individuals have higher odds to attend screening programs (45, 46). On the other hand, about the association between educational level and screening attendance, some studies found no significant association (47, 48), while others described a positive correlation between higher educational level and the attendance (49, 50). Higher household income is associated with higher attendance rate on screening (31, 50).

In addition to these, some studies have also identified other factors that have a positive or negative effect on participation in cervical cancer screening: the most common barriers were pain/discomfort; embarrassment; and time, the most commonly reported facilitators were: ease of making appointments; peace of mind; and fear of cancer/preventing serious illness (51).

## 5 Strengths and limitations

This study utilized data from the European Health Interview Survey (EHIS), which offers a representative sample of the adult population in Hungary. Although the same methodology was applied consistently across all three survey years, an aggregated dataset was employed for comparative analysis. Multiple logistic regression models were utilized to identify significant determinants of screening uptake, providing valuable insights for the design of targeted intervention strategies. Owing to the methodological design of the data collection process, the database includes only data from participants, with no information available for those who opted not to participate. The European Health Interview Survey (EHIS), as administered by Eurostat and the Hungarian Central Statistical Office (HCSO), did not differentiate between Type 1 and Type 2 diabetes mellitus. However Type 2 diabetes has higher prevalence, we assumed that the majority of respondents had type 2 diabetes. This limitation has been acknowledged, and results should be interpreted with this consideration in mind.

## 6 Conclusions

This study focused on the importance of cervical cancer screening participation in women with diabetes and highlights the benefits of screening within this population. In particular, it should be taken into account that the participation rate in screening is not homogeneous in terms of the population. In the case of participation in cervical cancer screening, it is lowest in those groups whose health-influencing factors are not very favorable (those with poor financial status, those with perceived poor health, those taking prescription medications). Therefore, the identification of groups with lower screening participation may be crucial in the context of the introduction of public health interventions aimed at improving the participation rate.

## Data Availability

The data analyzed in this study is subject to the following licenses/restrictions: The datasets of the European Health Interview Survey for this study are available upon request from the Hungarian Central Statistical Office (https://www.ksh.hu). Requests to access these datasets should be directed to Karolyne Tokaji, Karolyne.Tokaji@ksh.hu.
